# The feedback loop of ANKHD1/lncRNA MALAT1/YAP1 strengthens the radioresistance of CRC by activating YAP1/AKT signaling

**DOI:** 10.1038/s41419-022-04554-w

**Published:** 2022-02-02

**Authors:** Ping-an Yao, Yong Wu, Kui Zhao, Yecheng Li, Jianping Cao, Chungen Xing

**Affiliations:** 1 Department of General Surgery, Second Affiliated Hospital of Soochow University, Suzhou, 215004 China; 2 School of Radiation Medicine and Protection, Medical College of Soochow University, Suzhou, 215123 China; 3 State Key Laboratory of Radiation Medicine and Protection and Collaborative Innovation Center of Radiation Medicine of Jiangsu Higher Education Institutions, Soochow University, Suzhou, 215123 China

**Keywords:** Radiotherapy, Apoptosis, Double-strand DNA breaks

## Abstract

Innate radioresistance substantially limits the effectiveness of radiotherapy for colorectal cancer (CRC); thus, a strategy to enhance the radiosensitivity of CRC is urgently needed. Herein, we reported that ankyrin repeat and KH domain containing 1 (ANKHD1) serves as a key regulator of radioresistance in CRC. ANKHD1 was highly expressed in CRC tissues and was highly correlated with Yes-associated protein 1 (YAP1) in CRC. Our results first revealed that ANKHD1 knockdown could increase the radiosensitivity of CRC by regulating DNA-damage repair, both in vitro and in vivo. Furthermore, the interactive regulation between ANKHD1 or YAP1 and lncRNA MALAT1 was revealed by RIP and RNA pull-down assays. Moreover, our results also demonstrated that MALAT1 silencing can radiosensitize CRC cells to IR through YAP1/AKT axis, similar to ANKHD1 silencing. Taken together, we report a feedback loop of ANKHD1/MALAT1/YAP1 that synergistically promotes the transcriptional coactivation of YAP1 and in turn enhances the radioresistance of CRC by regulating DNA-damage repair, probably via the YAP1/AKT axis. Our results suggested that targeting the YAP1/AKT axis downstream of ANKHD1/MALAT1/YAP1 may enhance the radiosensitivity of CRC.

## Introduction

Colorectal cancer (CRC) is one of the most common malignant tumors, there are almost 900000 deaths caused by CRC annually, and CRC ranks as the fourth most deadly cancer in the world [1]. Neoadjuvant chemoradiotherapy followed by surgery is the standard treatment for locally advanced CRC [2]. Most patients show a reduction in tumor size after radiotherapy; however, only 15–20% of patients have a complete response to radiotherapy, some patients experience recurrence after radiotherapy [1], and the 5-year survival rate is less than 65% [3]. Resistance to radiotherapy is a main factor resulting in poor clinical outcomes. Therefore, it is critical to clarify the mechanism of radioresistance, which may provide new ways to improve the outcome of patients.

Ankyrin repeat and KH domain containing 1 (ANKHD1) is a multifunctional protein with multiple ankyrin repeats and a single KH domain. The presence of multiple ankyrin repeats allows ANKHD1 to function as a scaffolding protein that mediates interactions between proteins; ANKHD1 is also a RNA-binding protein (RBP) owing to the function of the KH domain [4]. Recently, many studies have shown that ANKHD1 is highly expressed in various tumors, acting as an oncoprotein and significantly shortening patient survival [5, 6]. Our early works revealed that ANKHD1 was upregulated in CRC and played an oncogenic role in the progression of CRC [7]. Nevertheless, the function of ANKHD1 in the regulation of radiosensitivity remains poorly defined.

Metastasis-associated lung adenocarcinoma transcript 1 (MALAT1) is a highly conserved long noncoding RNA (lncRNA). It was first defined in lung cancer as a metastasis-promoting factor [8]. The lncRNA MALAT1 has been widely studied, and accumulating evidence indicates that MALAT1 acts as an oncogene in the process of various cancers (e.g., prostate cancer, gastric cancer, and lung cancer) [9–11]. Several studies have reported that the expression of MALAT1 is related to tumor radioresistance [12], however, the mechanism remains unclear. The MALAT1 transcript has two fragments, the larger fragment stays in the nucleus and localizes to nuclear speckles, and the smaller fragment has the ability to move to the cytoplasm with unclear function [13]. Studies have shown that nuclear components of MALAT1 function as RBPs and interact with KH-domain-containing proteins [14]. Since ANKHD1 contains a KH domain, we speculated that ANKHD1 may be an interaction partner of MALAT1, coregulating the radiosensitivity of CRC.

The transcriptional regulator Yes-associated protein 1 (YAP1) is the central component of Hippo signaling [15], and YAP1 plays an oncogenic role in many cancers [16, 17]. As a transcription coactivator, YAP1 cooperates with transcription factors to bind to targeted DNA, thus mediating carcinogenic effects by activating downstream transcripts [18]. In this study, we found that ANKHD1 interacts with YAP1, which allows YAP1 to translocate into the nucleus and avoid degradation in the cytoplasm, enhancing the transcriptional activity of YAP1. An existing study indicated that YAP1-induced MALAT1 promotes epithelial–mesenchymal transition and angiogenesis in CRC [19], and the present study also revealed an interaction between ANKHD1 or YAP1 and MALAT1. Therefore, we speculate that there may be an interaction circuit among ANKHD1/MALAT1/YAP1, which may coordinately regulate the radiosensitivity of CRC.

In the present study, we found that elevated ANKHD1 expression was highly correlated with YAP1 and that ANKHD1 silencing increased the radiosensitivity of CRC cells. Mechanistically, we investigated the interactions of ANKHD1, MALAT1, and YAP1 and explored their roles in DNA-damage repair. Finally, we discovered a ANKHD1/MALAT1/YAP1 feedback loop that synergistically promotes radioresistance via YAP1/AKT axis-mediated DNA-damage repair. The results of this study may provide new ideas related to the ANKHD1/MALAT1/YAP1 loop for improving the radiosensitivity of CRC.

## Results

### ANKHD1 knockdown enhanced the radiosensitivity of CRC both in vitro and in vivo

**Fig. 1:**
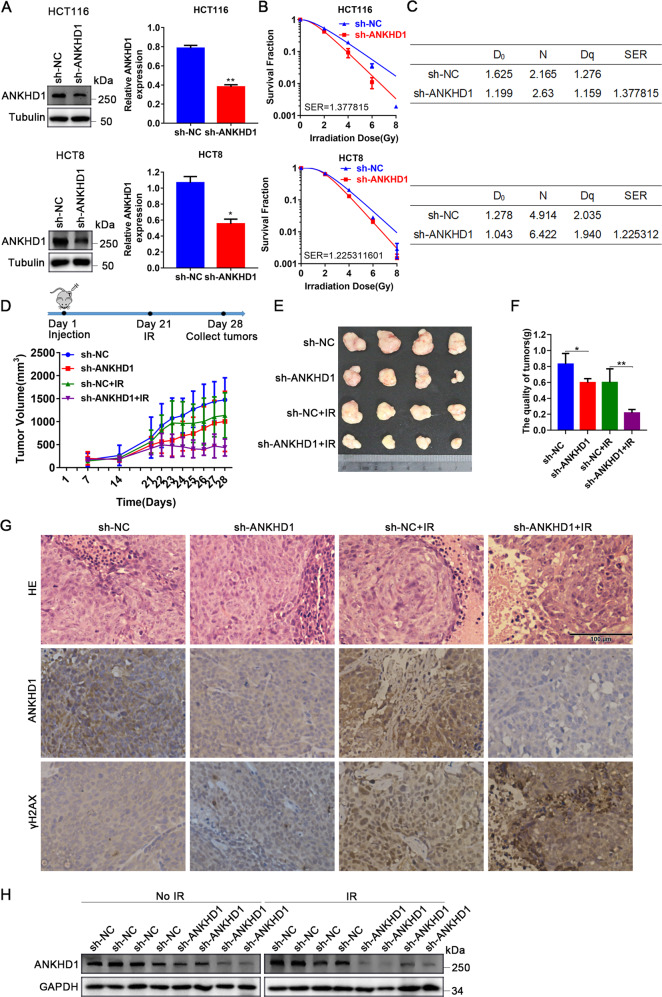
ANKHD1 knockdown enhanced the radiosensitivity of CRC both in vitro and in vivo. **A** ANKHD1 expression levels were tested by Western blot in HCT116 and HCT8 cells infected with lentivirus-targeting ANKHD1, the silencing efficiency was analyzed using Image J (*P < 0.05, **P < 0.01). **B** ANKHD1 silencing increased the radiosensitivity of HCT116 and HCT8 colorectal cancer cells (SER = 1.378 in HCT116, SER = 1.225 in HCT8). **C** The D_0_, N, Dq, and SER values of ANKHD1-silenced cells, the SER value was simulated using the multi-target single-hit model. **D**, **E** Tumor-growth curves and images of all tumors of each group (*n* = 5 and 4 tumors were observed). **F** The quality of each group tumors (*P < 0.05, **P < 0.01). **G** H&E staining of tumor section of all groups in this study, IHC was performed to test the expression of ANKHD1 and γH2AX in the tumor section of all groups in this study. **H** The expression of ANKHD1 in subcutaneous tumor tissues was detected by Western blot.

To investigate the ability of ANKHD1 to regulate the radiosensitivity of CRC, ANKHD1 depletion was performed in the CRC cell lines HCT116 and HCT8 using lentivirus (Fig. 1A). Clonogenic assay showed that ANKHD1 knockdown markedly increased radiosensitivity in both HCT116 and HCT8 cells, with sensitization-enhancement ratios (SERs) of 1.378 and 1.225, respectively (Fig. 1B, C). In addition, a significant difference in cell survival was observed when cells were exposed to 4 Gy IR (Fig. S1A).

CRC xenograft mouse model was established to further evaluate the radioresistance caused by ANKHD1 in vivo. As shown in Fig. 1D–F, the growth of ectopic tumors in the sh-ANKHD1 group was significantly inhibited post 10 Gy irradiation. HE staining of tumor sections showed that the volume of solid tumors was significantly reduced after irradiation, especially in the sh-ANKHD1 group (Fig. 1G). Decreased expression of ANKHD1 was observed in tumor sections from the sh-ANKHD1 group with or without IR, and increased γH2AX expression was observed post IR (Fig. 1G H). Together, these results revealed that ANKHD1 silencing promoted radiosensitivity both in vitro and in vivo.

### ANKHD1 silencing promoted IR-induced DNA double-strand breaks by inhibiting DNA-damage repair signaling

**Fig. 2:**
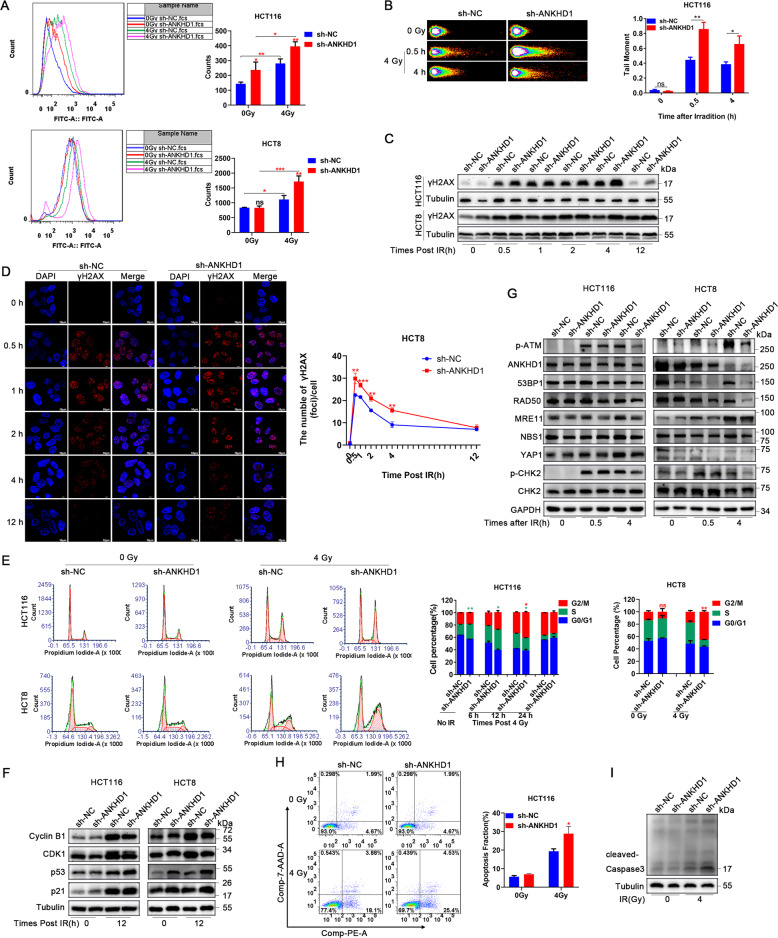
ANKHD1 silencing promoted IR-induced DNA double-strand breaks by inhibiting DNA-damage repair signaling. **A** The formation of ROS was detected by flow cytometry in HCT116 and HCT8 cells with or without IR (*P < 0.05, **P < 0.01). **B** A comet assay was conducted to detect DNA damage at 0, 0.5, and 4 h after 4 Gy IR, and the DNA-damage amount was quantified by measuring the comet-tail lengths (*P < 0.05). **C** Western blotting was used to detect γH2AX at 0, 0.5, 1, 2, 4, and 12 h post IR. **D** γH2AX foci were detected by immunofluorescence at 0, 0.5, 1, 2, 4, and 12 h post IR in CHT8 cells, and the γH2AX foci number was counted from more than 100 cells (**P < 0.01). **E** Flow cytometry was used to detect the distribution of the cell cycle; ANKHD1-silenced cells were arrested in G2/M phase at 12 h after 4 Gy IR (*P < 0.05). **F** The expression of cyclin B1, CDK1, p53, and p21 was detected by Western blotting at 0 and 12 h post IR in HCT116 and HCT8 cells. **G** The expression of p-ATM, 53BP1, RAD50, MRE11, NBS1, YAP1, and p-CHK2 was detected by Western blot at 0, 0.5 and 4 h post IR in HCT116 and HCT8 cells. **H** ANKHD1 silencing increased apoptosis at 48 h post IR in HCT116 cells (*P < 0.05). **I** ANKHD1 silencing increased the expression of cleaved-caspase 3 at 48 h post IR.

The formation of reactive oxygen species (ROS) is an early event post IR and is an indirect cause of DNA damage. Our results showed that deleting ANKHD1 significantly promoted IR-induced ROS formation in CRC cell lines (Fig. 2A). Interestingly, we noticed that ANKHD1 knockdown also promoted ROS formation in HCT116 cells without IR, which indicated that ANKHD1 may affect ROS generation via certain signaling pathways. IR-induced DNA double-strand breaks (DSBs) are the main cause of IR-induced cell death, which represents the radiosensitivity of certain types of cells [20]. Therefore, we explored whether ANKHD1 can affect IR-induced cellular DBSs. As shown in Fig. 2B, ANKHD1 depletion obviously increased HCT116 cell DSBs at 0.5 and 4 h after IR, with IR-induced tail moment reaching maximum levels at 0.5 h post IR in the sh-ANKHD1 group, represented as nearly 2-fold the length of the control group. Furthermore, prominently boosted IR-induced γH2AX foci formation was observed at 0.5 h post 4 Gy IR in the sh-ANKHD1 group by applying γH2AX immunofluorescence staining and protein-expression determination (Fig. 2C, D and Fig. S2A, B).

Once cells suffer DBSs, the DNA-damage response (DDR) is instantly triggered, represented as the activation of a cascade phosphorylation in the nucleus. ATM was rapidly activated by the MRE11/RAD50/NBS1 (MRN) sensor complex upon IR-induced DBSs [21], and then DNA-damage repair was achieved through the nonhomologous end-joining (NHEJ) or homologous-recombination (HR) pathway in the S and G2 phases. Cells with unrepaired DNA were prevented from entering mitosis. Our results showed that the percentage of cells in G2/M phase gradually increased and reached maximum levels at 12 h post IR, especially the ANKHD1-silencing group (Fig. 2E). To reveal the regulation of the cell cycle by ANKHD1 post IR, we detected the expression of p53, p21, cyclin B1, and CDK1, which are regulators of the G2/M phase checkpoint. ANKHD1 knockdown obviously inhibited cyclin B1 and CDK1 expression and increased p53 and p21 expression at 12 h post IR (Fig. 2F).

To gain insight into the molecular mechanism of DDR, cascade phosphorylation in the progression of DDR was determined. The results showed that ANKHD1 silencing inhibited the expression of the MRN (MRE11/RAD50/NBS1) complex, resulting in inhibition of ATM phosphorylation. The phosphorylation of the checkpoint protein CHK2 and the expression of 53BP1 were subsequently decreased (Figs. 2G and S2C). These results suggested that ANKHD1 silencing might inhibit DDR via both HR and NHEJ signaling, probably by suppressing the MRN complex. Apart from cell-cycle arrest, the accumulation of DNA damage ultimately induces cell death. The results of our flow-cytometry experiments indicated that ANKHD1 silencing enhanced cell death post 4 Gy IR (Fig. 2H), and we also observed high cleaved-caspase-3 expression in ANKHD1-silenced cells post IR (Fig. 2I). In summary, we suggested that ANKHD1 silencing promotes IR-mediated DNA damage and inhibits DNA-damage repair, ultimately leading to cell apoptosis, which contributes to the enhancement of radiosensitivity in CRC cells.

### MALAT1 knockdown enhanced radiosensitivity by inhibiting DDR in CRC cells

**Fig. 3:**
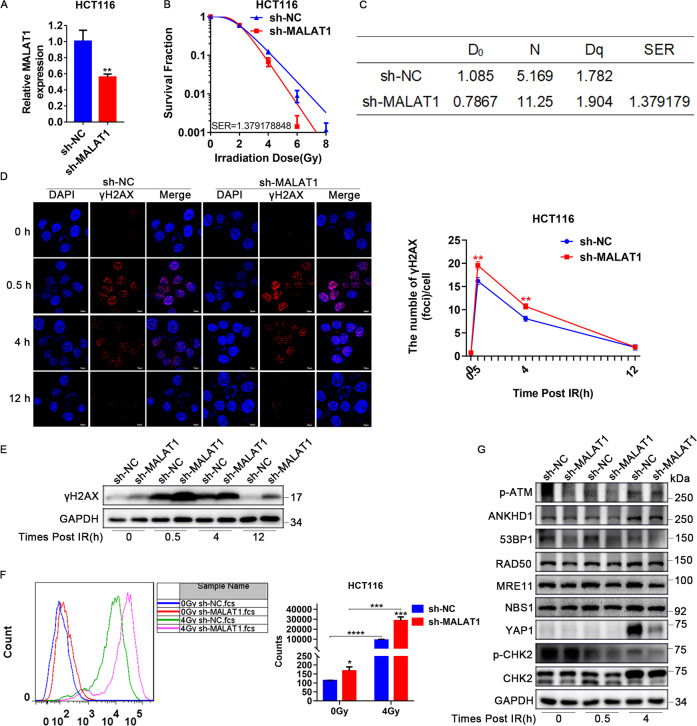
MALAT1 knockdown enhanced radiosensitivity by inhibiting DDR in CRC cells. **A** MALAT1 expression level was tested by qRT-PCR in HCT116 cells infected with lentivirus-targeting MALAT1 (**P < 0.01). **B** MALAT1 silencing decreased the survival fraction of HCT116 cells post IR, SER = 1.379. **C** The D_0_, N, Dq and SER values of MALAT1-silenced cells, the SER value was simulated using the multitarget single-hit model. **D** γH2AX foci were detected by immunofluorescence at 0, 0.5, 4, and 12 h post IR in CHT116 cell, and the number of γH2AX foci was counted in more than 100 cells (**P < 0.01). **E** The expression of γH2AX was tested at 0, 0.5, 4 and 12 h after 4-Gy IR. **F** The formation of ROS was tested by flow cytometry at 0.5 h after 4 Gy IR in MALAT1-silenced HCT116 cells (*P < 0.05, ***P < 0.001). **G** The expression of p-ATM, ANKHD1, 53BP1, RAD50, MRE11, YAP1, p-CHK2, and CHK2 was detected by Western blotting at 0, 0.5, and 4 h after 4 Gy IR in MALAT1-silenced cells.

To gain insight into the function of MALAT1, shRNA-mediated MALAT1-depleting HCT116 cells were constructed and verified by qRT-PCR assay (Fig. 3A). Clonogenic assays showed that MALAT1 knockdown significantly increased radiosensitivity, with SER = 1.379 (Figs. 3B, C and S1B). Western blot and IF assays both indicated that MALAT1 knockdown promoted IR-induced DSBs in CRC cells. For example, the γH2AX foci number reached maximum levels at 0.5 h post IR (Fig. 3D, E). DNA damage lasted until 12 h post IR in sh-MALAT1-group cells, while it was repaired at 12 h post IR in the control group (Fig. 3E). In addition to direct damage caused by IR, MALAT1 knockdown also induced DNA damage due to the formation of ROS with or without IR (Fig. 3F). Furthermore, the Western blot results showed that radiation could induce high expression of YAP1 at 4 h post IR, while this induction was weakened when MALAT1 was knocked down. MALAT1 silencing significantly inhibited the expression of the MRN complex, impaired the phosphorylation of ATM and CHK2, and inhibited 53BP1 expression (Fig. 3G). Together, these data indicated that MALAT1 silencing inhibited the DDR through both the HR and NHEJ pathway, by which MALAT1 silencing increased the radiosensitivity of CRC cells.

### The interaction between ANKHD1 and YAP1 stabilizes the transcriptional activity of YAP1

**Fig. 4:**
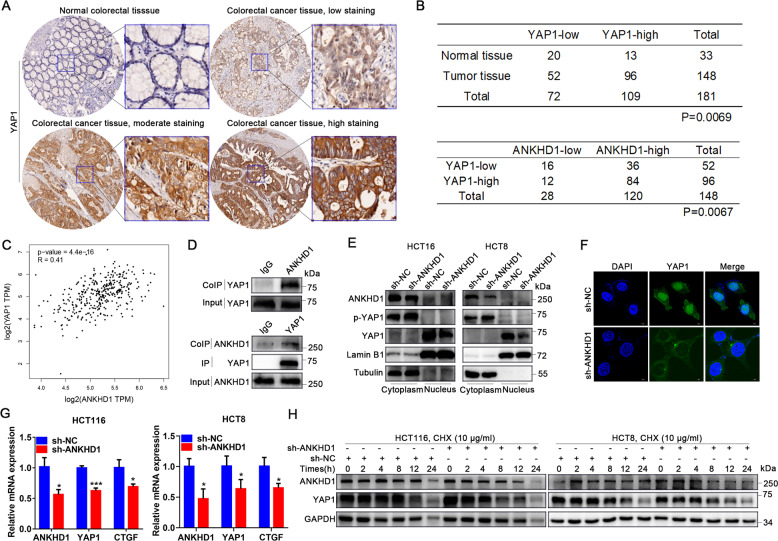
The interaction between ANKHD1 and YAP1 stabilizes the transcriptional activity of YAP1. **A** The expression of YAP1 was tested by IHC in colorectal cancer tissue and normal tissue, and the staining of YAP1 in tumors was divided into three levels: low staining, moderate staining, and high staining. **B** The chi-square test showed an obvious difference in YAP1 expression between colorectal tumors and normal tissue (top, *P* < 0.005), and the chi-square test showed a high correlation of the expression of ANKHD1 and YAP1 in colorectal tumors (bottom, *P* < 0.05). **C** The correlation between the expression of ANKHD1 and YAP1 was analyzed in CRC using the GEPIA database (*P* < 0.05). **D** Immunoprecipitation indicated that ANKHD1 could bind to YAP1 in HCT116 cells. **E** A nucleus-cytoplasm separation assay showed that ANKHD1 silencing decreased YAP1 expression in the nucleus and increased the phosphorylation of YAP1 in the cytoplasm. **F** The location of YAP1 was measured by immunofluorescence, indicating that ANKHD1 silencing inhibited the nuclear translocation of YAP1. **G** qRT-PCR was performed to detect the mRNA expression of ANKHD1, YAP1, and CTGF, and the data are presented as the mean ± SD (*P < 0.05, ***P < 0.001). **H** Western blot analysis showed that ANKHD1 silencing increased the degradation of ANKHD1 and YAP1 after CHX treatment (10 μg/ml).

Our previous research demonstrated that ANKHD1 was highly expressed in CRC tissue and promoted CRC cell proliferation, migration, and invasion by activating EMT via YAP1 [7]. To further determine the relevance of ANKHD1 and YAP1, we evaluated YAP1 protein expression in colorectal tumors as well as normal tissues by IHC on tissue microarrays. The staining of YAP1 was divided into three levels in colorectal tumor tissue: low staining, moderate staining, and strong staining (Fig. 4A). An obvious difference of YAP1 expression was observed between colorectal tumors and normal tissues (*P* = 0.0069): 39% (13 of 33) of normal colorectal tissue tested high expression of YAP1, whereas 65% (96 of 148) of tumors showed high YAP1expression (Fig. 4B, top). These data indicated that high YAP1 expression may be a predictor of tumors. In addition, upregulated ANKHD1 was observed in 81% (120 of 148) of colorectal tumors. A significant positive correlation was found between ANKHD1 and YAP1 in the tested colorectal tumors: 70% (84 of 120) of tumors with high expression of ANKHD1 also showed high YAP1 levels (Fig. 4B, bottom). Our results were in accordance with those of a correlation analysis based on GEPIA database (Fig. 4C).

In addition, the direct interaction between ANKHD1 and YAP1 was confirmed by CoIP assay (Fig. 4D). A nucleus–cytoplasm-separation assay revealed that ANKHD1 silencing inhibited YAP1 translocation into the nucleus and exacerbated the phosphorylation of YAP1 in the cytoplasm (Fig. 4E, F). These results indicated that ANKHD1 silencing might inhibit the transcriptional coactivation of YAP1. Next, the transcriptional activity of YAP1 was detected by testing the mRNA abundance of CTGF, a main downstream target of YAP1. As shown in Fig. 4G, ANKHD1 silencing markedly inhibited YAP1 and CTGF mRNA expression. Furthermore, the accelerated degradation of YAP1 under cyclohexamide (CHX) treatment was demonstrated in sh-ANKHD1 cells (Fig. 4H). Overall, these data indicated that the interaction between ANKHD1 and YAP1 stabilized each other in the cytoplasm, which ensured the nuclear accumulation and transcriptional coactivation of YAP1.

### The feedback loop of ANKHD1/MALAT1/YAP1 is responsible for YAP1 activity

**Fig. 5:**
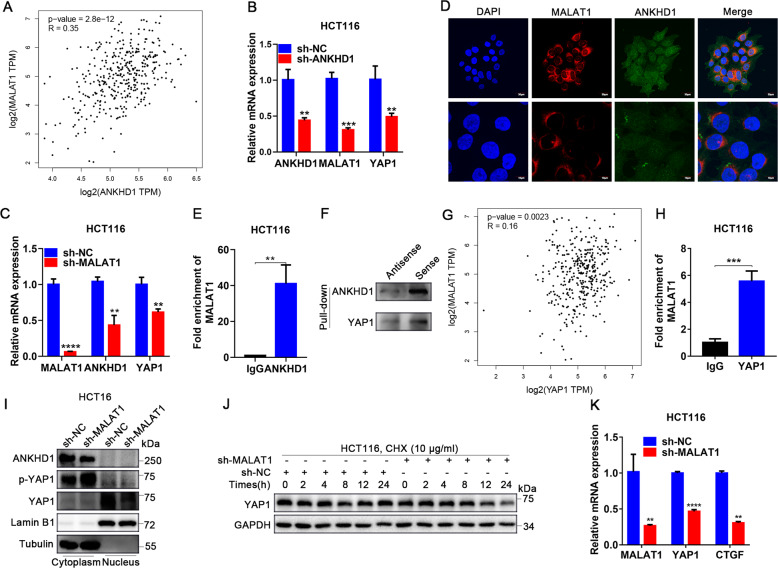
The feedback loop of ANKHD1/ MALAT1/YAP1 is responsible for YAP1 activity. **A** Correlation of the expression of ANKHD1 and MALAT1 was analyzed in colorectal cancer using the GEPIA database (*P* < 0.05). **B**, **C** qRT-PCR was performed to detect the mRNA expression of ANKHD1, MALAT1, and YAP1 in ANKHD1-silenced or MALAT1-silenced cells, and the data are presented as the mean ± SD (**P < 0.01, ***P < 0.001). **D** Immunofluorescence staining indicated the colocalization of ANKHD1 and MALAT1 in the cytoplasm. **E**, **H** RNA-immunoprecipitation assay showed the binding of ANKHD1 or YAP1 with MALAT1. qRT-PCR was used to detect the RNA level of MALAT1 in the precipitates, and data are presented as the mean ± SD (**P < 0.01, ***P < 0.001). **F** RNA pull-down experiment showed the interaction between ANKHD1 or YAP1 and MALAT1, biotin-labeled MALAT1 was incubated with HCT116 cell lysates, and the enriched ANKHD1 or YAP1 was detected by Western blotting. The antisense MALAT1 was used as control. **G** Correlation of the expression of MALAT1 and YAP1 was analyzed in colorectal cancer using the GEPIA database (*P* < 0.05). **I** A nucleus-cytoplasm separation assay showed that MALAT1 silencing decreased YAP1 expression in the nucleus and increased the phosphorylation of YAP1 in the cytoplasm. **J** Western blot analysis showed that MALAT1 silencing increased the degradation of YAP1 after treatment with CHX (10 μg/ml). **K** MALAT1 silencing inhibited the expression of YAP1, as well as CTGF downstream of YAP1. Data are presented as the mean ± SD (**P < 0.01, ***P < 0.001).

Quantitative proteomics revealed that ANKHD1 was an interacting protein of MALAT1, but the interaction between ANKHD1 and MALAT1 was not verified, especially their co-effect on radiosensitivity [22]. By applying bioinformatics analysis based on GEPIA database, MALAT1 expression was found to be closely related to ANKHD1 in CRC (Fig. 5A), which was verified by qRT-PCR (Fig. 5B, C). To further verify the interaction, the cellular localization of MALAT1 and ANKHD1 was examined by FISH combined with IF staining. MALAT1 and ANKHD1 were colocalized in the cytoplasm of CRC cells (Fig. 5D). Furthermore, the RIP assay showed an obvious enrichment of MALAT1 in anti-ANKHD1 with greater than 40-fold enrichment compared with anti-IgG (Fig. 5E). Reciprocally, RNA pull-down and Western blot analysis showed that ANKHD1 could bind with MALAT1 (Fig. 5F). These results indicated that MALAT1 physically associates with ANKHD1.

In addition, the positive correlation between MALAT1 and YAP1 was predicted in CRC based on GEPIA database (Fig. 5G), which was confirmed by qRT-PCR that the abundance of YAP1 mRNA was significantly decreased in MALAT1-silenced CRC cells (Fig. 5C). Moreover, RIP assay showed a significant enrichment of MALAT1 in anti-YAP1 with nearly 6-fold enrichment compared with anti-IgG (Fig. 5H). Conversely, RNA pull-down and Western blot analyses showed that YAP1 could bind with MALAT1 (Fig. 5F). Then, inhibited nuclear translocation of YAP1 and increased phosphorylation of YAP1 were detected in MALAT1-silenced CRC cells (Fig. 5I). Furthermore, protein-degradation assays indicated that MALAT1 silencing accelerated YAP1 degradation (Fig. 5J), and qRT-PCR results showed that MALAT1 silencing reduced the mRNA abundance of CTGF (Fig. 5K). These results suggested that MALAT1 silencing inhibited YAP1 transcriptional coactivation. Overall, we report an interaction loop of ANKHD1/MALAT1/YAP1 that synergistically regulates the transcriptional activity of YAP1.

### YAP1 is an important regulator of the DDR

**Fig. 6:**
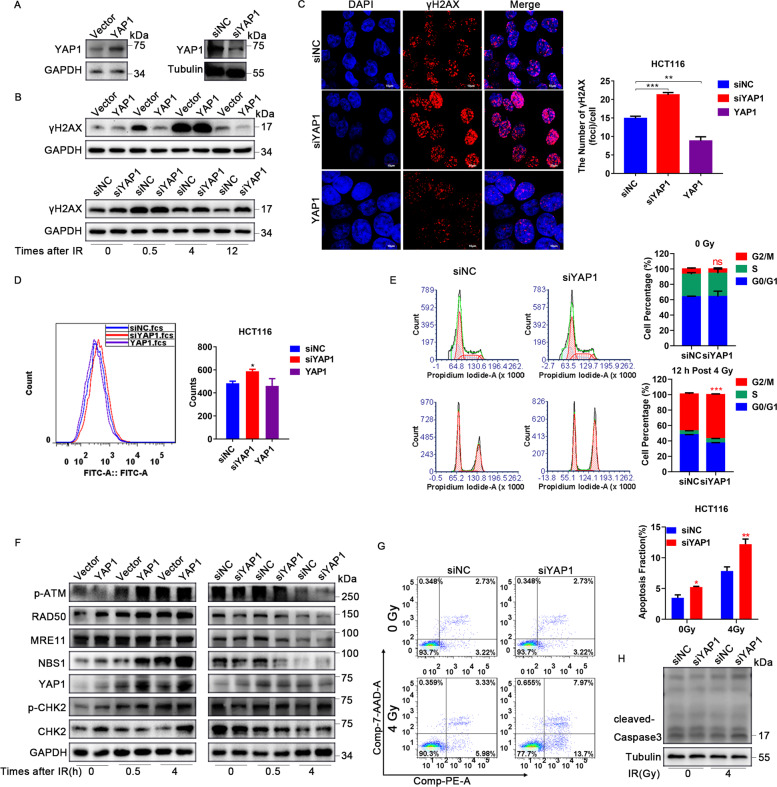
YAP1 is an important regulator of the DDR. **A** Western blotting was used to detect the expression of YAP1 in HCT116 cells transfected with overexpression plasmid or siRNA targeting YAP1. **B** The expression of γH2AX was detected by Western blotting at 0, 0.5, 4, and 12 h after 4 Gy IR in HCT116 cells with upregulated or downregulated expression of YAP1. **C** γH2AX foci were detected by immunofluorescence at 0.5 h post IR in HCT116 cells with upregulated or downregulated expression of YAP1, and the number of γH2AX foci was counted in more than 100 cells (**P < 0.01). **D** YAP1 expression affected the formation of ROS in HCT116 cells (*P < 0.01). **E** Downregulation of YAP1 induced cell arrest in G2/M phase at 12 h post IR (***P < 0.001). **F** The expression of p-ATM, RAD50, MRE11, NBS1, YAP1, p-CHK2, and CHK2 was tested by Western blot at 0, 0.5, and 4 h post IR in HCT116 cells with upregulated or downregulated expression of YAP1. **G** Downregulation of YAP1 increased apoptosis at 48 h post IR, and the data are presented as the mean ± SD (*P < 0.05, **P < 0.01). **H** Downregulation of YAP1 increased the expression of cleaved-caspase 3 at 48 h post IR.

As ANKHD1 and MALAT1 both interact with YAP1 and regulate its transcriptional activity, we wondered whether YAP1 is essential in ANKHD1 or MALAT1-mediated radioresistance in CRC. First, to explore the role of YAP1 in the DDR, YAP1 overexpression or knockdown HCT116 cells were constructed by transfecting a YAP1 overexpression plasmid or siRNA (Fig. 6A). Western blot assays showed that YAP1 overexpression can suppress IR-induced γH2AX, and vice versa (Fig. 6B). As shown in Fig. 6C, compared with the control group, more γH2AX foci were observed in YAP1-knockdown cells, in contrast, fewer γH2AX foci were detected in YAP1-overexpression cells. Next, significantly increased ROS was detected in YAP1-knockdown CRC cells, and vice versa (Fig. 6D). Then, the distribution of the cell cycle was tested, and YAP1 knockdown obviously increased cell arrest in G2/M phase post IR (Fig. 6E). To clarify the effect of YAP1 on DDR, the expression of the MRN complex and the checkpoint proteins ATM and CHK2 was evaluated at 0, 0.5, and 4 h post IR. Western blot results showed that YAP1 overexpression significantly increased the expression of the MRN complex and further activated ATM and CHK2; conversely, YAP1 knockdown had the opposite effects (Fig. 6F). Consistent with the results above, IR-induced cell apoptosis was found to be significantly increased in YAP1-knockdown CRC cells, as well as high level of cleaved-caspase 3 (Fig. 6G, H). These results provide strong evidence that YAP1 may play a central role in regulating the IR-induced DDR of CRC.

### YAP1/AKT axis is essential for ANKHD1/MALAT1-mediated radioresistance of CRC cells

**Fig. 7:**
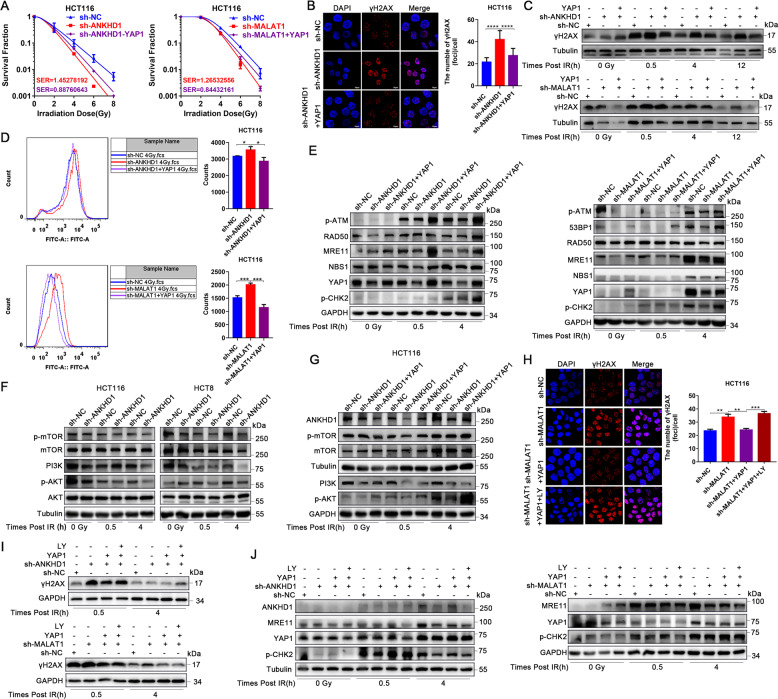
YAP1/AKT axis is essential for ANKHD1/MALAT1-mediated radioresistance of CRC cells. **A** Overexpression of YAP1 abrogated radiosensitivity in sh-ANKHD1 or sh-MALAT1 cells. **B**, **H** Overexpression of YAP1 reduced the large number of γH2AX foci induced by ANKHD1 silencing or MALAT1 silencing at 0.5 h post IR (**P < 0.01). **C** γH2AX was also tested by Western blotting at 0, 0.5, 4, and 12 h post IR. **D** Overexpression of YAP1 decreased the formation of ROS caused by ANKHD1 silencing or MALAT1 silencing at 0.5 h post IR (*P < 0.05). **E** Overexpression of YAP1 recovered the expression of p-ATM, 53BP1, RAD50, MRE11, NBS1, and p-CHK2 in ANKHD1-silenced cells or MALAT1-silenced cells after 0.5- and 4-Gy IR. **F** ANKHD1 silencing inhibited the activation of the PI3K/AKT signaling pathway with or without IR. **G** Overexpression of YAP1 reactivated the PI3K/AKT signaling pathway. **H**, **I** Treatment with the PI-3 kinase inhibitor LY294002 abrogated the effect of YAP1 on the expression of γH2AX in sh-ANKHD1 or sh-MALAT1 cells. **J** Western blot results showed that inactivation of AKT caused by LY294002 markedly decreased YAP1-induced upregulation of MRE11 and phosphorylation of the checkpoint protein CHK2 at 0.5 and 4 h post IR.

Furthermore, to verify whether YAP1 was essential for ANKHD1 or MALAT1-mediated radioresistance, rescue experiments were performed by overexpression of YAP1 in sh-ANKHD1 or sh-MAKAT1 cells. Clonogenic assays showed that ANKHD1 or MALAT1-silencing-mediated radiosensitivity was largely abrogated by YAP1 overexpression (Fig. 7A). With respect to radiosensitivity, CRC cells with ANKHD1 or MALAT1 silencing had significantly increased γH2AX foci-forming ability post IR, whereas YAP1 overexpression largely abrogated ANKHD1 or MALAT1-silencing-induced DNA damage in CRC cells (Fig. 7B, C, B: γH2AX foci at 0.5 h post IR). Interestingly, YAP1 overexpression also inhibited IR-induced ROS in sh-ANKHD1 or sh-MALAT1 CRC cells (Fig. 7D). Furthermore, YAP1 overexpression obviously reversed the inhibition of the MRN complex in ANKHD1- or MALAT1-silenced cells, which in turn reactivated the checkpoint proteins ATM and CHK2 at 4 h post IR (Fig. 7E). These results illustrated that YAP1, which functions as a regulator of the DDR, might be essential in ANKHD1- or MALAT1-mediated radioresistance in CRC cells.

A previous study reported that YAP promotes radioresistance through IGF2-mediated AKT activation [23]. Moreover, AKT promotes DSB repair by upregulating MRE11 expression post IR [24]. In our study, ANKHD1 silencing significantly inhibited the activation of the PI3K/AKT pathway (Fig. 7F); however, YAP1 overexpression reactivated AKT (Fig. 7G). Next, the PI-3 kinase inhibitor LY294002 was used to explore whether ANKHD1/MALAT1/YAP1 loop-mediated radioresistance required the activation of AKT. As shown in Fig. 7H, I, LY294002 abrogated the effect of YAP1 on DNA damage in sh-ANKHD1 or sh-MALAT1 cells, which implied that YAP1-decreased DNA damage might be the effect of downstream AKT activation. Next, we verified the role of AKT in the DDR regulated by ANKHD1 or MALAT1. Western blot results showed that inactivation of AKT caused by LY294002 markedly decreased YAP1-induced upregulation of MRE11 and phosphorylation of checkpoint protein CHK2 at 0.5 and 4 h post IR (Fig. 7J). In summary, these results indicated that the ANKHD1/MALAT1/YAP1 loop increased the DDR of CRC cells in a YAP1-dependent manner, and the activation of AKT downstream of YAP1 played an important role in this process.

## Discussion

Accumulating evidence has demonstrated that dysregulated ANKHD1 serving as an oncoprotein is associated in various malignant tumors [25, 26]. Our previous study indicated that ANKHD1 was highly expressed in CRC and that ANKHD1 promoted CRC cell proliferation, invasion, and migration by activating YAP1 [7]. In the present study, we revealed another potential effect of ANKHD1 in radiotherapy. This was the first report that ANKHD1 can affect radiosensitivity of CRC both in vitro and in vivo. The mechanistic data illustrated that ANKHD1 knockdown facilitated IR-induced ROS and nuclear DSBs in CRC cells, and inhibited the DDR signaling.

Structurally, ANKHD1 contains both ankyrin repeat and KH domain, which endow ANKHD1 with the ability to simultaneously regulate proteins and RNAs. Here, we revealed that ANKHD1 could interact with lncRNA MALAT1 and YAP1 simultaneously. MALAT1 is a widely expressed lncRNA with a length of 8000 nt that is abnormally expressed in numerous cancer types [13] and plays an important role in the proliferation, invasion, metastasis, and angiogenesis of bladder cancer and glioma [27, 28]. The main effect of MALAT1 is regulating alternative splicing and pre-mRNA splicing, besides, MALAT1 also interacts with RNA-binging proteins [29]. Here, we discovered that ANKHD1 functioned as a novel MALAT1-binding protein, which was verified by RIP and RNA pull-down assays. Besides the evidence of physical association between ANKHD1 and MALAT1, the colocalization of ANKHD1 and MALAT1 in the cytoplasm further strengthened the association between these molecules. Interestingly, there are two different transcripts of MALAT1, the larger one localizing on nuclear speckles. Recently, Zhao Y et al. reported that the nuclear-encoded MALAT1 was aberrantly enriched in the mitochondria of hepatoma cells [30]. As to the smaller one, which is regarded as MALAT1-associated small cytoplasmic RNA (mascRNA), remains unknown [13]. Herein, we illustrated the interaction between ANKHD1 and MALAT1, nevertheless, further studies should be carefully carried out to illustrate whether the interaction is related to mascRNA or the mitochondrial translocation of MALAT1.

In addition, we identified that ANKHD1 and MALAT1 both positively regulate the transcriptional activity of YAP1. YAP1 is a transcriptional coactivator and the main target of the Hippo pathway, and the oncogenic role of YAP1 has been widely investigated [31, 32]. In the present study, overexpressed YAP1 was verified in CRC patient samples and was positively correlated with ANKHD1. Herein, we tested the directly physical interaction between ANKHD1 and YAP1, suggesting that ANKHD1 directly affects YAP1 transcriptional activity rather than triggers the Hippo pathway, which is consistent with a previous report in lung cancer [26]. It has been reported that YAP1-induced MALAT1–miR-126-5p axis may regulate angiogenesis and EMT to promote metastasis in CRC [19]. Our RIP and RNA pull-down assays also uncovered the physical interaction between YAP1 and MALAT. Taken together, these results suggest that there might be an interactive loop among ANKHD1/MALAT1/YAP1 that synergistically regulates the transcriptional activity of YAP1.

ANKHD1 and MALAT1 both confer radioresistance in CRC, but the mechanism remains unclear. Our study was the first to uncover the coactivated roles of ANKHD1 and MALAT1 on YAP1. Several studies reported that YAP promotes radioresistance through activation of AKT and CDK6 [23, 33]. Hence, we speculated that YAP1 might exert a crucial role in ANKHD1- or MALAT1-mediated radioresistance. YAP1 silencing can increase the transcriptional activity of p53 and promote the expression of p21, which directly inhibits the expression of cyclin B1 and CDK1, ultimately resulting in G2/M-phase arrest [34], consistent with our results of ANKHD1-silencing cells. DNA damage recruits MRN complexes to activate the ATM/CHK pathway and initiates DNA-damage repair, which eventually promoted radioresistance [35]. Here, we observed that ANKHD1 or MALAT1 silencing inhibited the DDR by decreasing the transcriptional activity of YAP1. AKT promotes DSB repair by upregulating MRE11 expression [36], and the activation of AKT is crucial in YAP1-mediated radioresistance [23]. In accordance with these studies, we showed that ANKHD1 increased the activation of AKT in a YAP1-dependent manner. By applying a PI-3 kinase inhibitor, YAP1 overexpression failed to induce upregulation of MRE11 and resulted in deactivation of CHK2, which ultimately failed to trigger DNA-damage repair in sh-ANKHD1 or sh-MALAT1 cells, suggesting that AKT was a crucial effector in regulating the ANKHD1/MALAT1/YAP1-loop-mediated radioresistance of CRC cells.

**Fig. 8:**
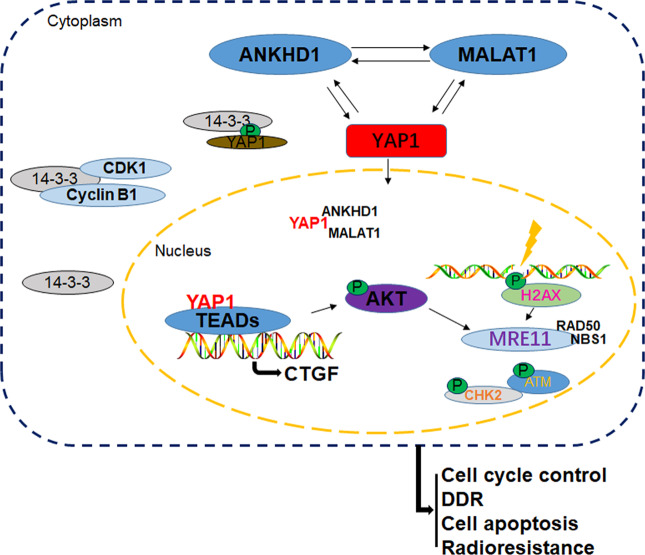
Overall schematic diagram. There is an ANKHD1/MALAT1/YAP1-feedback loop in CRC cells, which synergistically regulates the nuclear translocation of YAP1 and promotes the transcriptional coactivation of YAP1. Subsequently, YAP1-mediated activation of AKT increases the expression of the MRN complex and then activates the ATM/CHK2-checkpoint pathway, promoting DNA damage repair and ultimately resulting in radioresistance in CRC.

In conclusion, our present study first revealed that ANKHD1 expression can affect the radiosensitivity of CRC both in vitro and in vivo. We also proposed a potential interactive regulatory loop consisting of ANKHD1/MALAT1/YAP1 that might synergistically regulate the transcriptional activity of YAP1 and trigger the activation of AKT, which in turn influenced IR-induced DNA-damage repair in CRC (Fig. 8). Together, we provide a possible mechanism of the YAP1/AKT axis downstream of the ANKHD1/MALAT1/YAP1 loop, which might be a potential therapeutic target for comprehensive CRC therapy.

## Materials and methods

### Cell culture, lentivirus infection, and plasmid transfection

The human CRC cell lines HCT116 and HCT8 were purchased from Procell and were authenticated by STR profiling, there was no mycoplasma contamination (Wuhan, China). The cells were cultured in Dulbecco’s modified Eagle’s medium (DMEM) or RPMI-1640 (HyClone, Logan, UT, USA), respectively, supplemented with 10% fetal bovine serum (FBS) and 1% penicillin–streptomycin at 37 °C in a humidified atmosphere with 5% CO_2_. HCT116 and HCT8 cells were infected with lentivirus-mediated control shRNA or lentivirus-mediated shRNA targeting ANKHD1 or MALAT1 and named sh-NC, sh-ANKHD1, and sh-MALAT1, respectively. The lentivirus was purchased from Hanbio Biotechnology (Shanghai, China), and the details are listed in Supplementary Table S1. The YAP1-overexpression plasmid was obtained from Applied Biological Materials (Richmond, BC, Canada), and transfection was performed using Lipofectamine 3000 reagent (Invitrogen, Carlsbad, CA, USA). Subsequent experiments were performed at 48 h after transfection.

### Ionizing-radiation (IR) exposure

Cells were exposed to different dosages (0, 2, 4, 6, or 8 Gray (Gy)) of X-ray generated by a linear accelerator (RadSource, Suwanee, GA, USA) at a fixed dose rate of 1.15 Gy/min.

### Clonogenic assay

Cells are digested by trypsin containing 0.25% EDTA at the logarithmic growth phase and collected by centrifugation. After resuspension in the corresponding complete culture medium, the cells were seeded in 6-well plates at different densities (300, 300, 1000, 3000, and 5000 cells), and exposed to 0, 2, 4, 6, and 8 Gy, respectively, on the other day. After ionizing radiation, the cells were cultured for another 14 days until colony formation. Then, the cells were fixed and stained with crystal violet and counted the clone-formation rate. The radiation sensitivity-enhancement ratio (SER) was measured with a “single-hit multitarget” model.

### Flow-cytometry analysis

For cell-cycle distribution analysis, the cells were harvested at 12 h post 4 Gy IR and then fixed with 70% cold ethanol overnight. The cells were centrifuged and stained with propidium iodide (Beyotime Biotechnology, Shanghai, China) at 37 °C for 30 min and then determined by flow cytometry (BD Biosciences, Franklin Lakes, NJ, USA). For cell apoptosis analysis, cells were harvested at 48 h after 4-Gy IR, and apoptosis was measured using the 7-AAD/Annexin-V double-staining apoptosis kit (BD Biosciences, Franklin Lakes, NJ, USA) by flow cytometry. For ROS-generation analysis, cells were treated with the DCFH-DA probe (Beyotime Biotechnology, Shanghai, China) before 4-Gy IR. Another half hour of cultivation was needed after IR, and then the cells were harvested for detection of ROS by flow cytometry.

### Immunofluorescence staining (IF)

The cells were fixed in 4% paraformaldehyde for 10 min at the specified time points post irradiation. After permeabilization and blocking, cells were incubated with primary antibodies at room temperature and then incubated with secondary antibody for 1 h at room temperature. This was followed by conjugated staining with 4′,6‐diamidino‐2‐phenylindole (DAPI, Beyotime Biotechnology, Shanghai, China). Finally, the cells were observed by laser-scanning confocal microscopy (Olympus, Tokyo, Japan). The γH2AX foci were counted from the images from at least 100 cells from each group.

### Neutral-comet assay

The neutral-comet assay was conducted using a Trevigen Comet Assay Kit (Gaithersburg, MD, USA) according to the manufacturer’s instructions. Briefly, cells (5000/ml) were mixed with agarose and spread on slides quickly. The slides were immersed in lysis buffer and then subjected to electrophoresis in 1× neutral electrophoretic buffer. After electrophoresis, the slides were stained with SYBR Green (MESGEN, Shanghai, China). Finally, the images were captured by confocal microscopy (Olympus, Tokyo, Japan), and the tail moment was counted from at least 100 cells in each group.

### Quantitative real-time PCR (qRT-PCR)

Total RNA was extracted using TRIzol reagent (Invitrogen, Carlsbad, CA, USA), and complementary DNA (cDNA) was synthesized using 5× All-In-One MasterMix (Abm, Vancouver, Canada). Real-time PCR was performed using SYBR Green PCR Master Mix (QIAGEN, Germany) detected by an ABI PRISM 7500 Sequence Detection System (Applied Biosystems, Foster City, CA, USA). Relative quantitation was calculated by using RE = 2^(−ΔΔct)^, and GAPDH was used as a loading control. The primers used in our assay can be found in Supplementary Table S2.

### Immunohistochemistry

Tissue samples of 33 normal tissues and 148 colorectal cancer tissues were analyzed immunohistochemically. All surgically resected specimens were randomly obtained from patients diagnosed with CRC at the Second Affiliated Hospital of Soochow University from 2009 to 2014. The sections of tissue for IHC were incubated with antibodies against YAP1. The intensity of staining of the tissues was scored as follows: 0 (no staining), 1 (low staining, light yellow), 2 (moderate staining, yellowish brown), and 3 (strong staining, brown). An intensity score ≥2 was considered high expression, whereas intensity scores <2 were considered indicators of low expression. All slides were evaluated independently by two investigators blinded to the patient identities and clinical outcomes. This study was approved by the Ethics Committee of our hospital, and all patients provided written informed consent prior to enrolment.

### Western blotting and immunoprecipitation (IP)

Cells were lysed in RIPA buffer (Solarbio, Beijing, China) supplemented with the protease inhibitor PMSF (Solarbio, Beijing, China). Protein concentration was measured by a BCA protein quantitative kit (Beyotime Biotechnology, Shanghai, China), and 30 μg of protein from each sample was separated by sodium dodecyl sulfate–polyacrylamide-gel electrophoresis (SDS-PAGE) on 6–15% polyacrylamide gels and then transferred in 20% methanol buffer at 4 °C to Immobilon polyvinylidene difluoride (Millipore) membranes. Next, 5% BSA in TBST was used to block the membrane at room temperature for 1 h. Then, the membrane was incubated with primary antibodies at 4 °C overnight (antibodies used are listed in Supplementary Table S3). After three washes with TBST, the membrane was incubated with HRP-conjugated secondary antibody (Beyotime Biotechnology, Shanghai, China) at room temperature for 1 h. Next, the membrane was again washed three times and then visualized with an ECL detection kit (BIO-RAD, CA, USA) by FluorChem M (ProteinSimple, USA).

For the IP assay, sufficient antibody was added to 500 mg of protein and incubated with rotation overnight at 4 °C. The immunocomplexes were captured by adding 50 mL protein A/G agarose beads (Beyotime Biotechnology, Shanghai, China) and gently rotating for 3 h at 4 °C. Following centrifugation at 3000 g for 5 min at 4 °C, the supernatant was discarded. The precipitate was washed three times with ice-cold RIPA buffer and resuspended in 60 μL of 1× loading buffer. The immunocomplexes were dissociated from the beads by boiling for 5 min, and then the supernatant was subjected to Western blot analysis.

### CRC xenograft mouse-model construction and verification

Male Balb/c nude mice at 3–5 weeks of age were obtained from SLAC Laboratory Animal Company (Shanghai, China) and raised according to the guidelines of Soochow University for the use of experimental animals. The mice were randomized in a blinded fashion into two groups, then, 6 × 10^6^ HCT-116-sh-ANKHD1 cells or HCT116-sh-NC cells in 0.1 mL of PBS were injected subcutaneously into the right flanks of the nude mice. When the volume of the tumors reached 100 mm^3^, both groups of mice were randomized in a blinded fashion into two groups with or without irradiation at the tumor region (*n* = 5). Irradiation was conducted with a single dose of 10 Gy by linear accelerators. The tumor volumes were measured with an electronic Vernier caliper every day, using the following formula to calculate tumor volume (V = a*b^2^/2, a: the longest diameter; b: the shortest diameter). Then, the mice were sacrificed, and the tumors were isolated for hematoxylin and eosin (H&E) staining and immunohistochemistry as previously described [7]. All our animal experiments were approved by the Animal Care and Use Committee of Soochow University.

### Fluorescent in situ hybridization (FISH)

MALAT1 probes were designed and synthesized by RiboBio (Guangzhou, China). The probe signals were detected with a FISH Kit (RiboBio, Guangzhou, China) according to the manufacturer’s instructions. Briefly, HCT116 cells were fixed in 4% paraformaldehyde for 10 min. After prehybridization in PBS, the cells were hybridized at 37 C for 30 min in hybridization solution that contained FISH probe mix. Then, cell nucleus underwent counterstaining by utilizing DAPI (Beyotime, China). Images were captured using a laser-scanning confocal microscopy (Olympus, Tokyo, Japan).

### RNA immunoprecipitation (RIP)

Cellular proteins from HCT116 cells were extracted with RIP lysis buffer supplemented with protease-inhibitor cocktail and RNase inhibitor. Fifty microliters of magnetic beads and 5 µg of the antibody of interest and IgG control were incubated with rotation for 30 min at room temperature. Next, 100 µL of the RIP lysate and each bead–antibody complex were incubated with rotation overnight at 4 °C in RIP-immunoprecipitation buffer. Then, the coprecipitated RNAs were extracted with TRIzol reagent, and the copy number of MALAT1 in the RNA elute was analyzed by qRT-PCR.

### In vitro transcription and RNA pulldown

The templates for in vitro transcription were obtained from PCR. The primer containing the T7 promoter of MALAT1 was purchased from Sangon Biotech (Shanghai, China), and the sequences of the primers are listed in Supplementary Table S4. In vitro transcription was conducted using a TranscriptAid T7 High Yield Transcription Kit (Thermo Fisher Scientific, Waltham, MA, USA) following the manufacturer’s instructions, and the integrity and size of the synthesized RNAs were evaluated using agarose-gel electrophoresis.

RNA pull-down experiments were conducted using a Pierce™ Magnetic RNA–Protein Pull-Down Kit (Thermo Fisher Scientific, Waltham, MA, USA). In brief, cell lysates were prepared using IP lysis buffer, and the concentration was ensured to be more than 2 mg/mL. The target RNA was labeled with biotin using a Thermo Scientific Pierce RNA 3′ Desthiobiotinylation Kit. Next, the labeled RNA was incubated with streptavidin magnetic beads for 30 min at room temperature with agitation. Then, the bead–RNA complex and Master Mix of RNA-protein binding reaction were incubated for 60 min at 4 °C with rotation. Finally, the eluted interacting proteins were separated by 6% SDS-PAGE, and primary antibodies against ANKHD1 and YAP1 were used to detect the interaction with MALAT1.

### Statistical analysis

Data are shown as the mean ± SD of three independent experiments analyzed by Student’s *t*-test. Correlation analysis of proteins of interest in colorectal tumor tissue was performed with Pearson’s chi-square test. Two-tailed *P-*values < 0.05 were considered to indicate statistical significance. Statistical analyses were performed with GraphPad Prism 7.00 (GraphPad Software Inc., San Diego, USA) (*P < 0.05, **P < 0.01, ***P < 0.001).

## Supplementary information

Supplemental Material

aj-checklist

## Data Availability

The data that support the findings of this study are available from the corresponding author upon reasonable request.
