# Supplementary material for: The feedback loop of ANKHD1/lncRNA MALAT1/YAP1 strengthens the radioresistance of CRC by activating YAP1/AKT signaling
Source: Cell Death Dis. 2022 Feb 2;13(2):103. doi: 10.1038/s41419-022-04554-w (PMC8810793; doi:10.1038/s41419-022-04554-w)
Supplement: Supplementary file 1 — Supplemental Material [file 41419_2022_4554_MOESM1_ESM.docx]

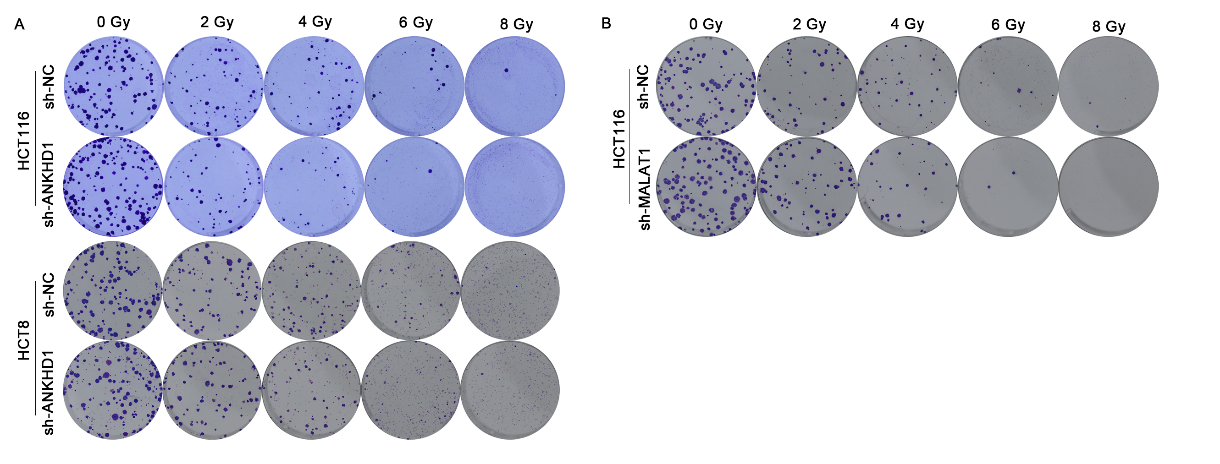
Supplementary Figure S1

**Supplementary Figure S1.** **Representative images of colony formation**. (A) Representative images of colony formation of ANKHD1-silencing group. (B) Representative images of colony formation of MALAT1-silencing group.


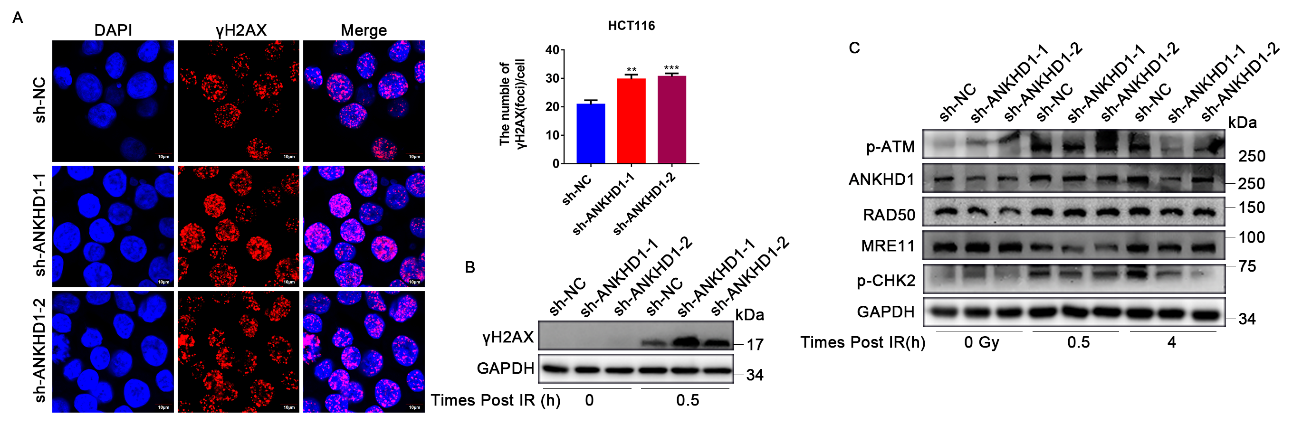
Supplementary Figure S2

**Supplementary Figure S2.** **ANKHD1 silencing promoted IR-induced DNA double-strand breaks and inhibited DNA damage repair signaling**. (A) γH2AX foci were detected by immunofluorescence at 0.5 h post IR in CHT8 cell, and the γH2AX foci number was counted from more than 100 cells (** *P* < 0.01, *** *P* < 0.001). (B) The expression of γH2AX was detected by Western blot, ANKHD1 silencing promoted γH2AX expression at 0.5 h post IR. (C) The expression of p-ATM, ANKHD1, RAD50, MRE11, and p-CHK2 was detected by Western blot at 0, 0.5 and 4 h post IR in HCT116 cell, ANKHD1 silencing inhibited the activation of DNA damage repair signaling.

Supplementary Table S1. Interfering sequences against ANKHD1, MALAT1 and YAP1

| Name | Sequence |
| --- | --- |
| sh-NC | TTCTCCGAACGTGTCACGTAA |
| sh-ANKHD1 | GCACTACTCTTAGCACAAGGA |
| sh-MALAT1 | AGATGAAGTTTGAGGTGGAAA |
| siNC | UUCUCCGAACGUGUCACGU |
| siYAP1 | CUGCCACCAAGCUAGAUAA |

Supplementary Table S2. Sequences of the DNA primers for qRT-PCR

| Name | Sequence (5’-3’) |
| --- | --- |
| ANKHD1 | Forward: CCTGCTTGGAACCCTATGATAAA  Reverse: CGTGCCAGGCCAAATCTG |
| MALAT1 | Forward: GGGTGTTTACGTAGACCAGAACC  Reverse: CTTCCAAAAGCCTTCTGCCTTAG |
| YAP1 | Forward: CGCTCTTCAACGCCGTCA  Reverse: AGTACTGGCCTGTCGGGAGT |
| CTGF | Forward: CAGCATGGACGTTCGTCTG  Reverse: AACCACGGTTTGGTCCTTGG |
| GAPDH | Forward: CATGAGAAGTATGACAACAGCCT  Reverse: AGTCCTTCCACGATACCAAAGT |

Supplementary Table S3. List of Antibodies used in this research.

| Name | Brand | Catalog No. | Dilution ratio |
| --- | --- | --- | --- |
| ANKHD1 | Abcam | Ab117788 | 1:1000 |
| YAP1 | Abcam | Ab52771 | 1:1000 |
| p-YAP1 | Abcam | Ab76252 | 1:1000 |
| γH2AX | Abcam | ab26350 | 1:1000 |
| p-ATM | BOSTER | BM4008 | 1:1000 |
| 53BP1 | Abcam | ab172580 | 1:1000 |
| MRE11 | Proteintech | 10744-1-AP | 1:1000 |
| NBS1 | Proteintech | 55025-1-AP | 1:1000 |
| RAD50 | CST | 3427 | 1:1000 |
| p-CHK2 | Beyotime | AC508 | 1:1000 |
| CHK2 | Beyotime | AF2020 | 1:1000 |
| PI3K | Affinity | AF6241 | 1:1000 |
| p-AKT | CST | 4060 | 1:1000 |
| AKT | CST | 4691 | 1:1000 |
| p-mTOR | CST | 5536 | 1:1000 |
| mTOR | CST | 2972 | 1:1000 |
| Lambin 1 | Abcam | Ab133741 | 1:1000 |
| p53 | Proteintech | 10442-1-AP | 1:1000 |
| p21 | CST | 2947S | 1:1000 |
| Cyclin B1 | Proteintech | 55004-1-AP | 1:1000 |
| CDK1 | Proteintech | 10762-1-AP | 1:1000 |
| Cleaved-Caspase3 | Immunoway | YM3431 | 1:1000 |
| Tubulin | Beyotime | AT819 | 1:1000 |
| GAPDH | Beyotime | AF5009 | 1:1000 |

Supplementary Table S4. Sequences for in vitro transcription of sense and antisense

MALAT1.

| Name | Sequence (5’-3’) |
| --- | --- |
| Sense | Forward: taatacgactcactatagggATGCAAGAAACATTCCAAACAAGCAACAG  Reverse:  AAATATACCGGTCCTGAAGACAGATTAGTAGTCAAAGCA |
| Antisense | Forward:  taatacgactcactatagggCCTGAAGACAGATTAGTAGTCAAAGCA  Reverse: AAATATACCGGTATGCAAGAAACATTCCAAACAAGCAACAG |
